# Coronary calcium scoring potential of large field-of-view spectral photon-counting CT: a phantom study

**DOI:** 10.1007/s00330-021-08152-w

**Published:** 2021-07-13

**Authors:** Niels R. van der Werf, S. Si-Mohamed, P. A. Rodesch, R. W. van Hamersvelt, M. J. W. Greuter, S. Boccalini, J. Greffier, T. Leiner, L. Boussel, M. J. Willemink, P. Douek

**Affiliations:** 1 Department of Radiology, University Medical Center Utrecht, Utrecht, The Netherlands; 2 Department of Radiology & Nuclear Medicine, Erasmus University Medical Center, Rotterdam, The Netherlands; 3 Louis Pradel Cardiology Hospital, Hospices Civils de Lyon, Lyon, France; 4 Univ Lyon, INSA-Lyon, Université Claude Bernard Lyon 1, UJM-Saint Etienne, CNRS, Inserm, CREATIS UMR 5220, U1206, Lyon, France; 5 Department of Radiology, University of Groningen, University Medical Center Groningen, Groningen, The Netherlands; 6 Department of medical imaging, Medical Imaging Group, Univ Montpellier, CHU Nimes, 2415 Nimes, EA France; 7 Department of Radiology, Stanford University School of Medicine, Stanford, CA USA

**Keywords:** X-ray computed tomography, Calcium, Coronary vessels, Imaging phantoms

## Abstract

**Objective:**

The aim of the current study was, first, to assess the coronary artery calcium (CAC) scoring potential of spectral photon-counting CT (SPCCT) in comparison with computed tomography (CT) for routine clinical protocols. Second, improved CAC detection and quantification at reduced slice thickness were assessed.

**Methods:**

Raw data was acquired and reconstructed with several combinations of reduced slice thickness and increasing strengths of iterative reconstruction (IR) for both CT systems with routine clinical CAC protocols for CT. Two CAC-containing cylindrical inserts, consisting of CAC of different densities and sizes, were placed in an anthropomorphic phantom. A specific CAC was detectable when 3 or more connected voxels exceeded the CAC scoring threshold of 130 Hounsfield units (HU). For all reconstructions, total CAC detectability was compared between both CT systems. Significant differences in CAC quantification (Agatston and volume scores) were assessed with Mann-Whitney U tests. Furthermore, volume scores were compared with the known CAC physical.

**Results:**

CAC scores for routine clinical protocols were comparable between SPCCT and CT. SPCCT showed 34% and 4% higher detectability of CAC for the small and large phantom, respectively. At reduced slice thickness, CAC detection increased by 142% and 169% for CT and SPCCT, respectively. In comparison with CT, volume scores from SPCCT were more comparable with the physical volume of the CAC.

**Conclusion:**

CAC scores using routine clinical protocols are comparable between conventional CT and SPCCT. The increased spatial resolution of SPCCT allows for increased detectability and more accurate CAC volume estimation.

**Key Points:**

• *Coronary artery calcium scores using routine clinical protocols are comparable between conventional CT and spectral photon-counting CT.*

• *In comparison with conventional CT, increased coronary artery calcium detectability was shown for spectral photon-counting CT due to increased spatial resolution.*

• *Volumes scores were more accurately determined with spectral photon-counting CT.*

**Supplementary Information:**

The online version contains supplementary material available at 10.1007/s00330-021-08152-w.

## Introduction

Spectral photon-counting computed tomography (SPCCT) is a novel emerging technology within the field of X-ray diagnostic radiology [1–7]. This technology employs energy discriminating photon-counting detectors (PCDs) to detect individual photons in more than 2 energy bins. Due to high photon flux in CT, small-pixel detectors are required to allow for individual photons to be counted without pulse pile-up effects [8–10]. In turn, the smaller PCD pixels result in superior spatial resolution in comparison with standard conventional energy integrating detector (EID) CT, which can be a major benefit for the assessment of coronary artery calcifications (CAC) [5, 11–14].

CAC is traditionally quantified on CT using the Agatston methodology (e.g., 120 peak kilovolt (kVp) acquisition; 3-mm slice thickness reconstruction) [15]. Quantification with Agatston scores is recommended by several guidelines to evaluate risk assessment for coronary artery disease [16–18]. The increased in-plane spatial resolution of SPCCT may result in reclassification of risk categories, as partial volume effects are decreased [19]. Especially small- and low-density coronary calcifications might not be resolved on the current EID CT system. This can potentially lead to the erroneous conclusion of a zero Agatston score, and correspondingly a misclassification to the lowest risk category. With the increased in-plane spatial resolution of SPCCT, the certainty of zero Agatston scores and Agatston score reproducibility can both potentially be increased. Through-plane increased spatial resolution will result in the same advantages, when data is reconstructed at small slice thickness. Furthermore, Agatston scores resulting from larger or higher density CAC can be impacted by this increased spatial resolution as well because of reduced blooming artefacts.

In addition to an increase in spatial resolution, SPCCT also decreases the impact of electronic noise. By setting the lowest energy bin threshold just above the electronic noise signal, the majority of noise can be successfully filtered out [1, 20]. This effect reduces the resulting total image noise [21–23]. This feature can potentially be used to acquire and reconstruct data at reduced slice thicknesses, so that both in-plane and through-plane CAC detection can be increased.

Because differences in Agatston scores between CT systems with EID or PCD elements are largely unknown the aim of the current study was twofold. First, the CAC scoring potential of SPCCT in comparison with conventional EID CT for routine clinical protocols was assessed. Second, the potential for improved CAC detection and quantification at reduced slice thickness will be assessed for SPCCT in comparison with EID CT.

## Materials and methods

### Phantom

An anthropomorphic (cardio) thoracic CT phantom (QRM Thorax, QRM GmbH) in combination with two different cardiac inserts was used. These inserts were a D100 insert and a cardiac calcification insert (CCI, QRM GmbH). Both inserts include cylindrical calcifications composed of hydroxyapatite (HA) powder. The D100 phantom contains 100 small calcifications of different sizes (ranged from 0.5 to 2.0 mm) and densities (ranged from 90 to 540 mgHAcm^-3^) and was used for the assessment of calcification detectability [24]. The CCI insert consists of nine calcifications with three different amounts of HA (200, 400, and 800 mgHAcm^-3^) and three different lengths and diameter (1.0, 3.0, and 5.0 mm) for each amount of HA. Additionally, to evaluate the effect of patient size, acquisitions were performed with and without a fat tissue-equivalent extension ring (QRM-Extension ring, QRM) simulating a small and large-sized patient, respectively [25].

### Acquisition and reconstruction parameters

Data acquisition was performed on two CT systems from one manufacturer: a dual-layer CT (DLCT) (IQon Spectral CT, Philips Healthcare) and a clinical spectral photon-counting CT (SPCCT) prototype (SPCCT, Philips Healthcare). The DLCT system was equipped with EID, while the SPCCT system was equipped with novel PCD [26].

Both devices were equipped with the same X-ray source and had the same source-to-isocenter and source-to-detector distances. Apart from the X-ray detection technology, the size of the detector pixels at iso-center was different between both systems, with 0.625 × 0.625 mm for EID and 0.275 × 0.275 mm for PCD. Further technical details concerning the prototype system and its performances are provided in previous studies [27, 28].

For both aims of the current study, routine clinical CAC scoring protocols were used for data acquisition and reconstruction (Table 1). For SPCCT, acquisition and reconstruction parameters were based on DLCT protocols recommended by the manufacturer. For the second aim, raw data were reconstructed at several combinations of slice thicknesses and increments, to assess the potential of improved detectability and quantification for both CT systems (Table 1). To counteract increased image noise at reduced slice thickness, several iterative reconstruction (IR) levels (iDose^4^ algorithm, Philips Healthcare) were added. Each scan was repeated five times, with manual repositioning between each scan (2-mm translation, 2 degrees rotation).

**Table 1:** Acquisition and reconstruction parameters for all used systems for the CAC scoring potential at routine clinical protocols

Parameter	DLCT	SPCCT
CT system	IQon	SPCCT
Technique	Sequential	Sequential
Tube voltage [kVp]	120	120
Tube current time product [mAs]	Small phantom: 40 Large phantom: 80	Small phantom: 40 Large phantom: 80
Automatic exposure correction	Off	Off
Focal spot	Standard	Small^1^
Collimation [mm]	64 × 0.625	64 × 0.275
Energy bin threshold [keV]	Not applicable	30 (lower) / 120 (upper)^1^
Field of view [mm]	220	220
Rotation time [s]	0.27	0.33
Slice thickness—increment [mm]	- 0.67–0.67 1.0–0.5 1.0–1.0 3.0–1.5 3.0–3.0	0.67–0.335 0.67–0.67 1.0–0.5 1.0–1.0 3.0–1.5 3.0–3.0
Reconstruction kernel	IQon-Std-B	SPCCT-Std-B^2^
Reconstruction matrix [pixels]	512 × 512	512 × 512
Reconstruction [iDose level]	0 / 3 / 5	0 / 3 / 5 ^3^
Repetitions	5	5

SPCCT was operated in conventional imaging mode, with only 2 thresholds to either suppress electronic noise (lower threshold) or to suppress pile-up counts (upper threshold)Despite differences in detector element size, reconstruction kernel, and reconstruction algorithm for SPCCT, reconstruction parameters for SPCCT were optimized by the manufacturer to get comparable results as with DLCT

The small focal spot is the only available option for the current clinical SPCCT prototype

### Analysis

#### General

Agatston scores were determined from the resulting reconstructed images using a previously validated, in-house developed Python script (Python version 3.7) [29]. To discriminate calcium-containing voxels from background material, a calcium scoring threshold of 130 HU was used. In addition, in line with the vendor-specific implementation for the Agatston score, a minimum connected area of 0.5 mm^2^ was used to include a group of voxels in the Agatston score of a specific calcification. For the used combination of field-of-view (220 mm) and reconstruction matrix (512 × 512), this results in a minimum of three connected voxels. In order to compare CAC quantification with physical volume, the volume score was also determined using the same in-house developed Python script [30].

In addition to the CAC scores, several image quality metrics were determined. First, mean HU values and noise levels (standard deviation (SD)) of the background material were calculated. Second, mean HU values of the largest calcifications of the CCI insert (5 mm diameter and length) were calculated and compared between both CT systems for the routine clinical protocol. Third, signal-to-noise ratios (SNR) were determined for these same calcifications and reconstructions. SNR was calculated as:

$$ SNR=\frac{CAC\ {HU}_{mean}}{Background\ {HU}_{SD}} $$

where CAC HU is the mean attenuation of the CAC, and Background HU_SD_ is the SD of the mean attenuation of the background. Fourth, contrast-to-noise ratios (CNR) were also determined for these calcifications and reconstructions. CNR were calculated as:

$$ CNR=\frac{CAC\ {HU}_{mean}- Background\ {HU}_{mean}}{Background\ {HU}_{SD}} $$

with Background HU_mean_ the mean of the attenuation of the background. And fifth, a background Agatston score (BAS) was evaluated for the D100 insert, whereby an Agatston score was calculated in the CAC containing slices with the CAC themselves automatically masked, resulting in a BAS score based on only noise [29]. CAC scores for slices with nonzero BAS were excluded, as it was unknown if actual CAC was measured, or if noise led to an Agatston score (Supplemental Figure 1).

#### Detectability (D100)

Detectability, assessed with the D100 insert, was defined as the ability to determine an Agatston score for a calcification for at least four out of the five repetitions. An Agatston was determined for a calcifications if at least three adjacent (horizontally or vertically) voxels were above the 130HU threshold. For the routine CAC protocol, detectability was assessed using previously described visibility curves [24]. The potential of CAC detection for both CT systems at reduced slice thickness was assessed with the number of detected calcifications.

#### Quantification (CCI)

For quantification of CAC, evaluated with the CCI insert, median CAC scores and range were calculated from the five repeated measurements. Because DLCT images could not be reconstructed at 0.67-/0.34-mm slice thickness/increment, comparison between SPCCT and DLCT scores was not possible for this slice thickness and increment. Comparisons with physical volume (98.2 mm^3^) were performed for the volume scores obtained with both CT systems.

### Statistical analysis

Mean HU, SNR, and CNR were compared between DLCT and SPCCT using a Mann-Whitney U signed-rank test, with a significance level of *p* < 0.05. Routine CAC protocol agreement between DLCT and SPCCT for Agatston scores was assessed using the Bland-Altman plots [31]. Differences in CAC quantification potential between DLCT and SPCCT at reduced slice thickness were assessed on the largest calcifications (5 mm diameter and length). For each combination of slice thickness and increment, CAC scores were compared with the reference (DLCT, reconstructed IR level 0) using a Mann-Whitney U signed-rank test, with a significance level of *p* < 0.05.

All statistical analysis were performed with SPSS version 27 (IBM SPSS Statistics).

## Results

### Image quality

**Fig. 1:**
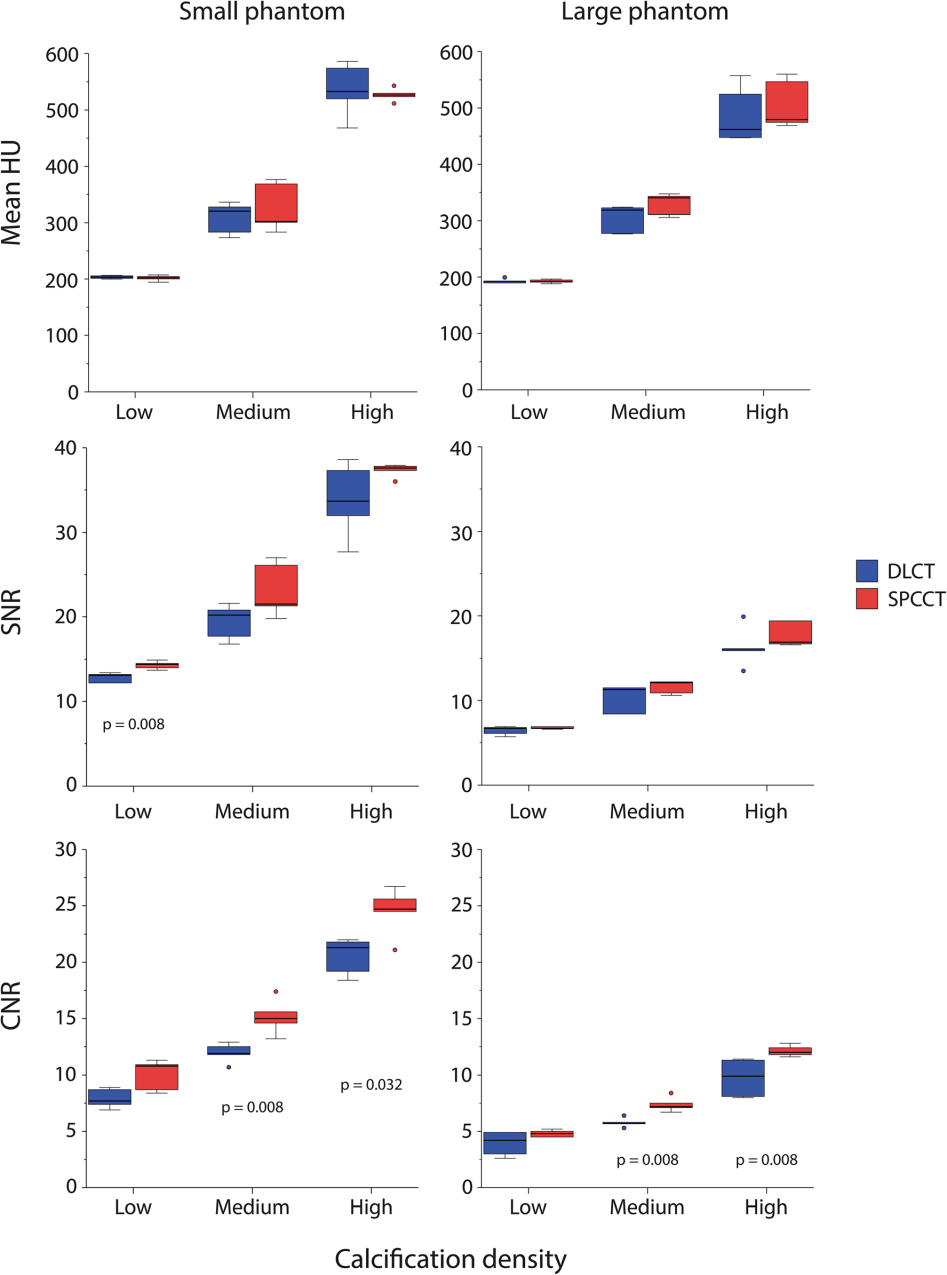
Mean HU, SNR, and CNR for the small and large phantom for the large (5 mm diameter and length) calcifications in the CCI insert for both dual-layer CT (DLCT) and spectral photon-counting CT (SPCCT)

Background mean CT number and image noise for both phantoms sizes and CT systems is shown in Table 2 for routine clinical protocols (3/3 mm slice thickness/increment, iDose level 0). Mean image noise was lower for SPCCT in comparison with DLCT, and for the small phantom size in comparison with the large phantom. Mean HU values and SNR for the largest calcifications of the CCI insert were comparable (*p* > 0.05) between both CT systems (Fig. 1). Only low-density CAC resulted in significantly different (*p* = 0.008) SNR between both CT systems. A significant increase (*p* < 0.05) in CNR for SPCCT was shown for the medium and high-density CAC. SNR and CNR were, in general, higher for the small phantom size for both CT systems.

**Table 2:** Background mean CT number (median (range)) and image noise (median (range)) for both phantom sizes and both CT systems for routine clinical protocols (3/3 mm slice thickness/increment, iDose level 0

CT system	Phantom size	Mean	Noise
DLCT	Small	35.9 (35.7–38.6)	15.4 (15.2–16.9)
	Large	49.3 (40.7–82.8)	28.8 (27.7–33.1)
SPCCT	Small	32.6 (32.3–33.0)	14.1 (13.9–14.3)
	Large	27.9 (27.1–28.3)	28.4 (28.1–28.8)

### Detectability (D100)

#### Routine CAC protocols

For routine CAC protocols (3/3 mm slice thickness/increment, iDose level 0), representative images for the D100 insert and detectability curves are shown in Supplemental Figure 2 and Supplemental Figure 3, respectively. In comparison with DLCT, more CAC were detected with SPCCT for the small phantom. This effect decreased for increased phantom dimensions.

#### CAC potential at reduced slice thickness

The percentage of detected CAC, with a total of 500 calcifications (five repetitions of D100 insert) as the denominator, is presented in Table 3. In comparison with 3-mm slice thickness and increment, detection of CAC increased, as expected, with overlapping slices and reduced slice thickness for both DLCT and SPCCT. For DLCT, detection increased by 142% from 12.8 to a maximum of 31% detected calcifications for reconstructions with 1-mm slice thickness, 0.5-mm slice increment, and IR level 0. At these reconstruction settings, SPCCT CAC detection was even 39% higher. SPCCT CAC detection increased by 169% from 17 to a maximum of 46% detected calcifications for reconstructions with 0.67-mm slice thickness, 0.335-mm slice increment, and IR level 3.

**Table 3:** Percentage of detected calcifications, with a total of 500 calcifications (five repetitions of D100 insert) as the denominator, for all combinations of slice thickness, slice increment, phantom size, and IR level, for both DLCT and SPCCT. Boldface entries indicate that a system has detected a higher number of calcifications compared to the other system for the same acquisition and reconstruction parameters. Italicized entries indicate that the number of detected calcifications is equal, while entries in bold italics indicate that less calcifications are detected by that system

CT system	Phantom size	IR level	Slice thickness/slice increment [mm]					
			3/3	3/1.5	1/1	1/0.5	0.67/0.67	0.67/0.335
DLCT	Small	0	***12.8%***	***17.2%***	***29.4%***	***31.0%***	***10.0%***	n/a
		3	***12.6%***	***16.6%***	***27.2%***	***28.8%***	***26.2%***	n/a
		5	***12.6%***	***16.4%***	***27.2%***	***27.6%***	***25.2%***	n/a
	Large	0	***14.8%***	***15.6%***	***0.0%***	***0.0%***	*0.0%*	n/a
		3	**14.4%**	**14.6%**	1.0%	5.6%	0.0%	n/a
		5	12.6%	13.0%	18.8%	19.4%	0.4%	n/a
SPCCT	Small	0	**17.2%**	**21.4%**	**40.6%**	**43.0%**	**45.4%**	**43.8%**
		3	**17.0%**	**20.8%**	**38.4%**	**39.4%**	**44.4%**	**46.2%**
		5	**16.6%**	**20.2%**	**36.2%**	**37.2%**	**41.8%**	**44.2%**
	Large	0	**15.4%**	**16.4%**	**0.8%**	**1.8%**	*0.0%*	**0.0%**
		3	***13.8%***	***14.4%***	**32.4%**	**19.0%**	**2.2%**	**0.0%**
		5	**12.8%**	**13.6%**	**28.0%**	**23.0%**	**27.2%**	**25.2%**

### Quantification (CCI)

#### Routine CAC protocols

**Fig. 2:**
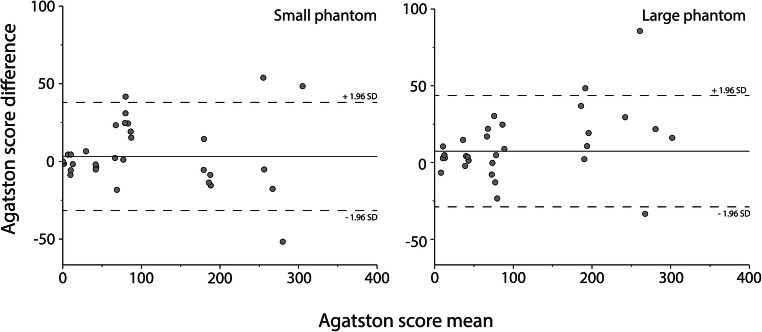
Bland-Altman plots for routine CAC protocols for the small (left) and large (right) phantom, comparing dual-layer CT (DLCT), and spectral photon-counting CT (SPCCT) Agatston scores. A positive difference indicates a higher Agatston score for DLCT

Agreement in CAC scores for the CCI insert between DLCT and SPCCT for routine CAC protocols is shown in Fig. 2. For the small phantom, the mean ± SD difference in Agatston score between both systems was very small at 3.2 ± 17.7. This difference in the Agatston score was slightly higher for the large phantom, at 7.4 ± 13.5. Differences increased with increasing Agatston scores.

#### CAC potential at reduced slice thickness

**Fig. 3:**
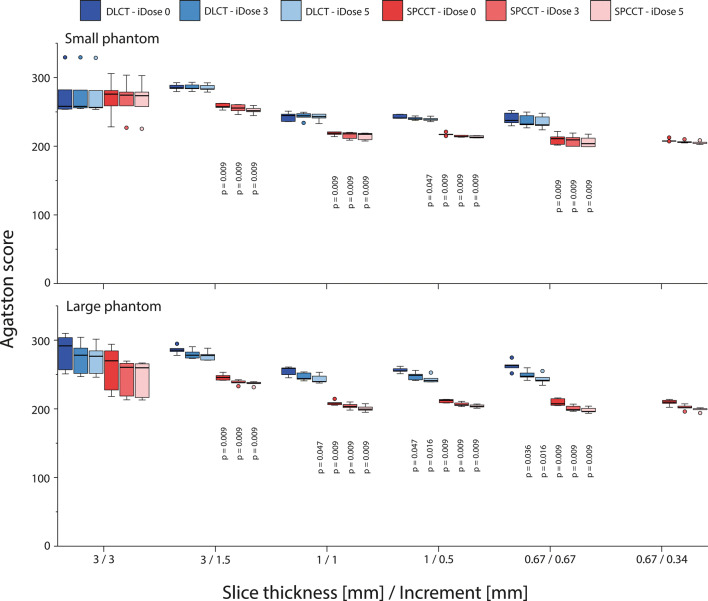
Agatston scores of the large calcification (5 mm diameter and length) with high density (800 mgHAcm^-3^), for acquisitions at different combinations of slice thickness and increment, reconstructed with different levels of IR, on both spectral photon-counting CT (SPCCT) and dual-layer CT (DLCT). Results are shown for the small (upper) and large (lower) phantom. For each combination of slice thickness and increment, *p* values from significant differences in comparison with the reference (DLCT and iDose 0) are indicated

**Fig. 4:**
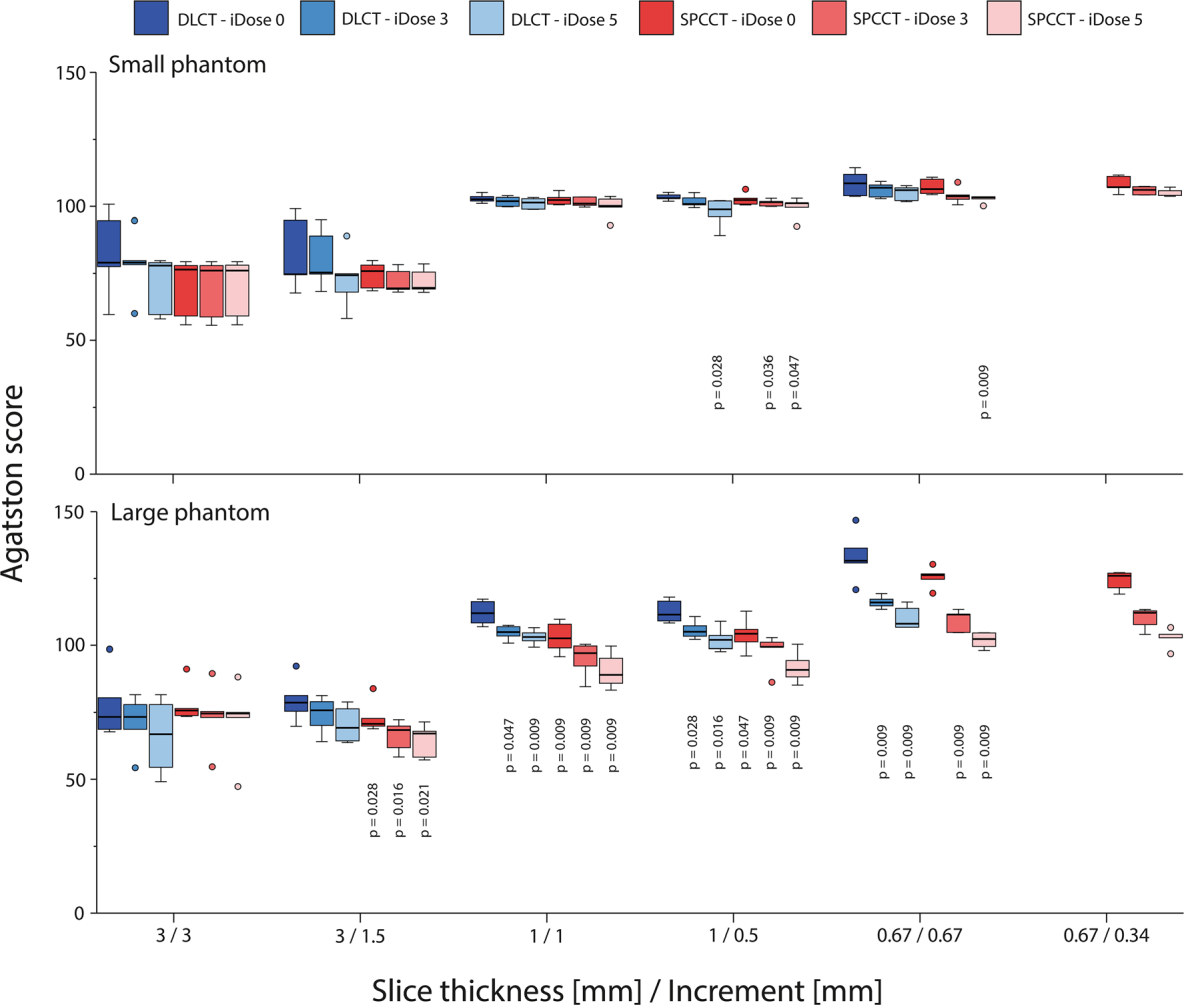
Agatston scores of the large calcification (5 mm diameter and length) with low density (200 mgHAcm^-3^), for acquisitions at different combinations of slice thickness and increment, reconstructed with different levels of IR, on both spectral photon-counting CT (SPCCT) and dual-layer CT (DLCT). Results are shown for the small (upper) and large (lower) phantom. For each combination of slice thickness and increment, *p* values from significant differences in comparison with the reference (DLCT and iDose 0) are indicated

High-density CAC Agatston scores showed significant differences (*p* < 0.05) between DLCT and SPCCT for almost all combinations of slice thickness and increment, irrespective of applied IR level or patient size (Fig. 3). Low-density CAC Agatston scores for the large phantom again show significant differences (*p* < 0.05) between DLCT and SPCCT (Fig. 4). However, Agatston scores were comparable for the small phantom size, when appropriate IR levels were applied.

**Fig. 5:**
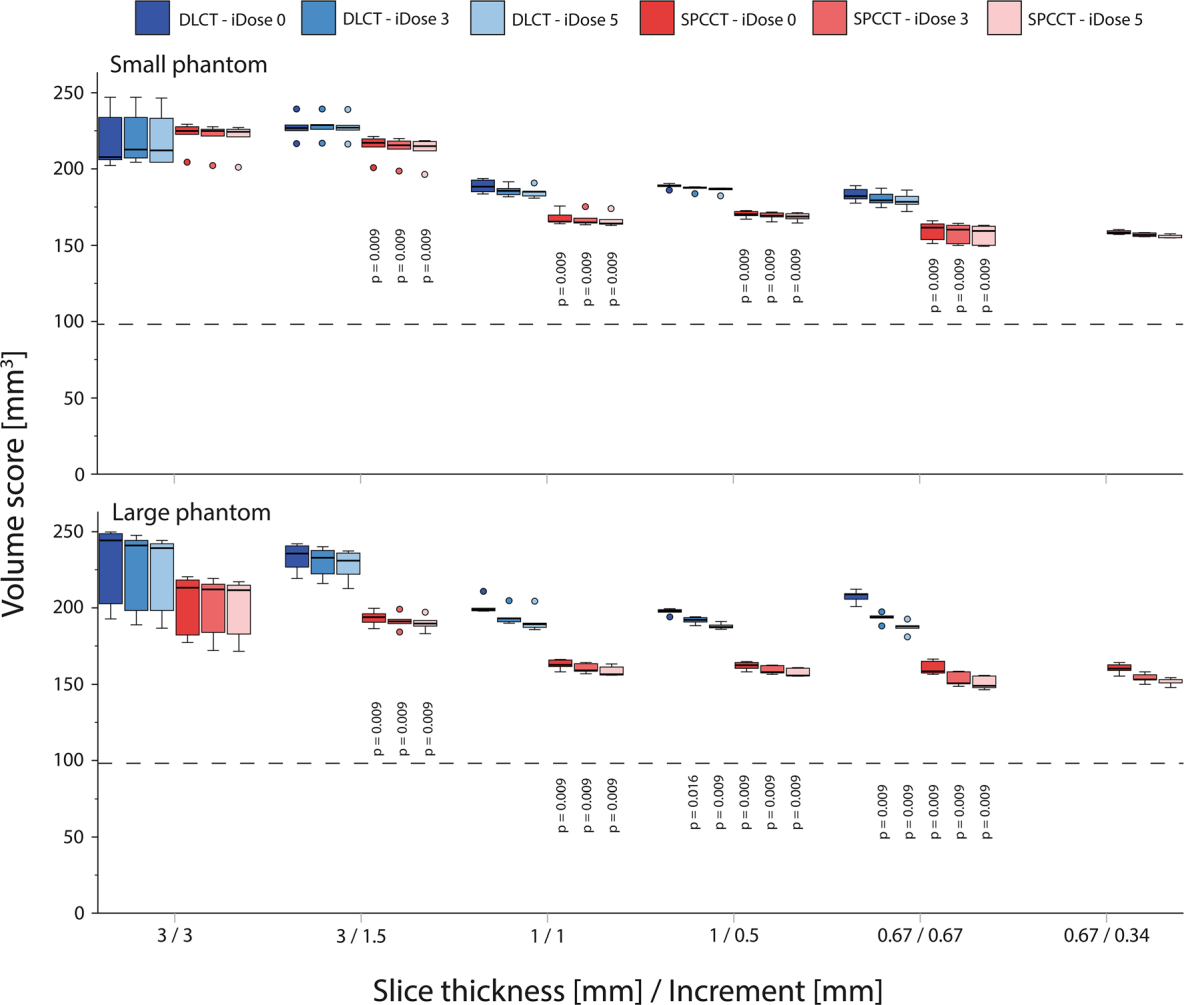
Volume scores of the large calcification (5 mm diameter and length) with high density (800 mgHAcm^-3^), for acquisitions at different combinations of slice thickness and increment, reconstructed with different levels of IR, on both spectral photon-counting CT (SPCCT) and dual-layer CT (DLCT). Results are shown for the small (upper) and large (lower) phantom. For each combination of slice thickness and increment, *p* values from significant differences in comparison with the reference (DLCT and iDose 0) are indicated. The dashed line indicates the physical volume of the calcification (98.2 mm^*3*^)

**Fig. 6:**
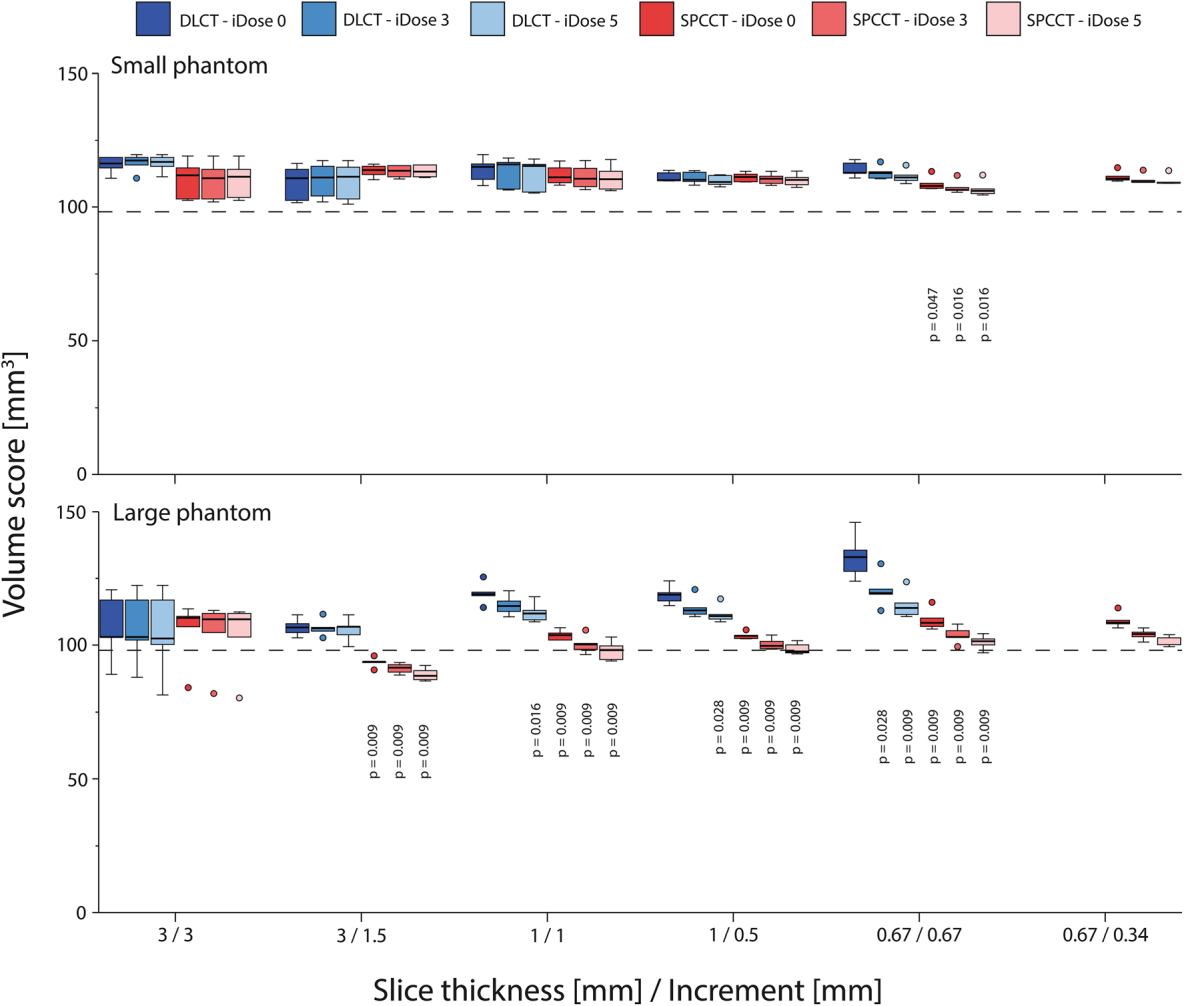
Volume scores of the large calcification (5 mm diameter and length) with low density (200 mgHAcm^-3^), for acquisitions at different combinations of slice thickness and increment, reconstructed with different levels of IR, on both spectral photon-counting CT (SPCCT) and dual-layer CT (DLCT). Results are shown for the small (upper) and large (lower) phantom. For each combination of slice thickness and increment, *p* values from significant differences in comparison with the reference (DLCT and iDose 0) are indicated. The dashed line indicates the physical volume of the calcification (98.2 mm^*3*^)

Volume scores showed similar trends as described above for the Agatston score (Figs. 5 and 6). When compared to the physical volume, high-density volume scores showed large overestimations (up to 150%) for all reconstructions. These overestimations decreased at smaller slice thicknesses because of reduced partial volume and blooming artefacts. For all reconstructions, overestimation of physical mass was smaller for SPCCT than for DLCT. Low-density volume scores showed better agreement with physical volume. For the large phantom, physical volume was overestimated by DLCT at reduced slice thickness due to noise effects.

## Discussion

In the present study, we found that SPCCT Agatston scores are comparable with conventional DLCT Agatston scores for routine CAC protocols. Furthermore, we found SPCCT to be more sensitive for the detection of CAC at reduced slice thickness acquisitions. Finally, we demonstrated that CAC quantification with SPCCT at reduced slice thickness using volume scores was more accurate than DLCT when compared to the actual physical volume of CAC.

Agatston scores are inherently associated with calcification density, due to the maximum voxel-based weighting factor. In addition, blooming artefacts, including partial volume artefacts, further increase the apparent size of medium and high-density CAC [32]. Also, very small calcifications might potentially be missed due to partial volume effects. This is clinically important because small- or low-density CAC may be more vulnerable compared to large or high-density CAC [33]. One solution to reduce blooming and partial volume artefacts is to increase spatial resolution. In the current study, we have shown that the effect of this increased spatial resolution is only minor for clinical CAC protocols, where only the in-plane resolution was improved, while the slice thickness was still set at 3 mm. This resulted in comparable CAC scores for these protocols on both scanners. For reduced slice thickness and/or overlapping slices, however, significant differences between DLCT and SPCCT were shown. For low-density CAC, the blooming artefact is inherently small. However, for the high-density calcification, reduced blooming artefacts resulted in more accurate CAC scores because of smaller deviations between the volume score and physical CAC volume for SPCCT. Furthermore, increased spatial resolution of SPCCT resulted in increased detectability of small or low-density calcifications for SPCCT. Finally, CAC visualization, as determined with SNR and CNR, increased for SPCCT due to reduced image noise in comparison with DLCT. This may be also the effect of a more important energy weighting of the lower energy photons due to the energy-resolving capabilities of the PCDs compared to the EIDs [1]. Altogether, our results are in line with a recent study by Symons et al, who also showed improved CAC CNR for a different SPCCT system, in comparison with conventional EID CT [23].

The strength of our study is that we systematically evaluated CAC scoring potential of SPCCT for routine and reduced slice thickness and slice increment, which provides a basis for future research and potential clinical application. In combination with the key findings of previous studies using SPCCT in the cardiovascular field, this modality is an exciting and promising tool for coronary artery disease with potential great expectations for patient management [6, 12, 34, 35]. Our study also has some limitations. First, we used a non-commercial SPCCT system for our evaluation. Second, we used a static anthropomorphic phantom. Despite the fact that the linear attenuation coefficients of the phantoms were in line with human materials at the used tube potential (120 kVp), a phantom does not completely simulate an actual human, with all internal organs. Also, coronary motion was not taken into account. Third, increased noise levels for reduced slice thickness or increased phantom size resulted in BAS > 0. With this, the possibility to assess CAC detectability was reduced, as it was unclear if a group of voxels above the CAC threshold contained CAC or noise. CAC detectability could therefore potentially be further increased, at the cost of increased radiation dose. Finally, current volume grid parameters were limited to the specifications of the used DLCT. Future studies can assess further improvements for SPCCT, such as other field-of-view and reconstruction matrix combinations, or increased IR strengths as recently reported [27].

In conclusion, CAC scores using routine clinical protocols are comparable between conventional CT and SPCCT. The increased spatial resolution of SPCCT allows for increased detectability and more accurate CAC volume estimation at reduced slice thickness.

## Supplementary Information

ESM 1 (DOCX 753 kb)

## Abbreviations

BAS: Background Agatston score
CAC: Coronary artery calcification
CNR: Contrast-to-noise ratio
DLCT: Dual-layer CT
EID: Energy integrating detector
HA: Hydroxyapatite
IR: Iterative reconstruction
PCD: Photon counting detector
SD: Standard deviation
SNR: Signal-to-noise ratio
SPCCT: Spectral photon-counting CT

## Declarations

### Guarantor

The scientific guarantor of this publication is Philippe Douek.

### Statistics and biometry

No complex statistical methods were necessary for this paper.

### Methodology

• prospective

• experimental

• performed at one institution
