# Supplementary material for: Coronary calcium scoring potential of large field-of-view spectral photon-counting CT: a phantom study
Source: Eur Radiol. 2021 Jul 13;32(1):152–62. doi: 10.1007/s00330-021-08152-w (PMC8660747; doi:10.1007/s00330-021-08152-w)

# Supplemental

Supplemental Figure 1 Illustration of impact of nonzero BAS on the ability to detect CAC. Both images show the slice of the D100 insert with the largest and most dense CAC. Left image is SPCCT with small phantom, reconstructed with 3 mm slice thickness, 3 mm slice increment and IR level 0. Image on the right is DLCT with the large phantom, reconstructed with 0.67 mm slice thickness, 0.67 mm slice increment and IR level 0


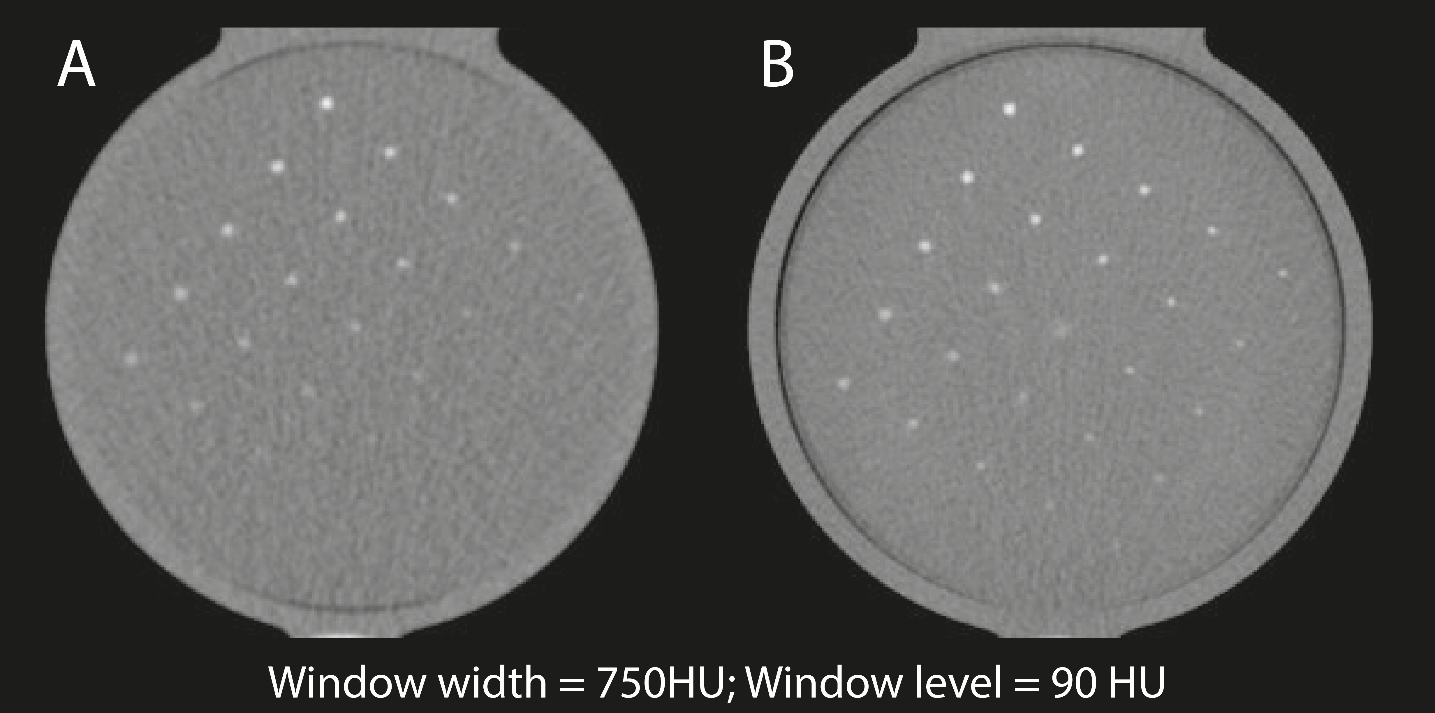


Supplemental Figure 2 Representative images of the D100 insert in combination with the small phantom for dual-layer CT (DLCT) and spectral photon-counting CT (SPCCT), as indicated. Window width and level were 750 and 90 HU, respectively.


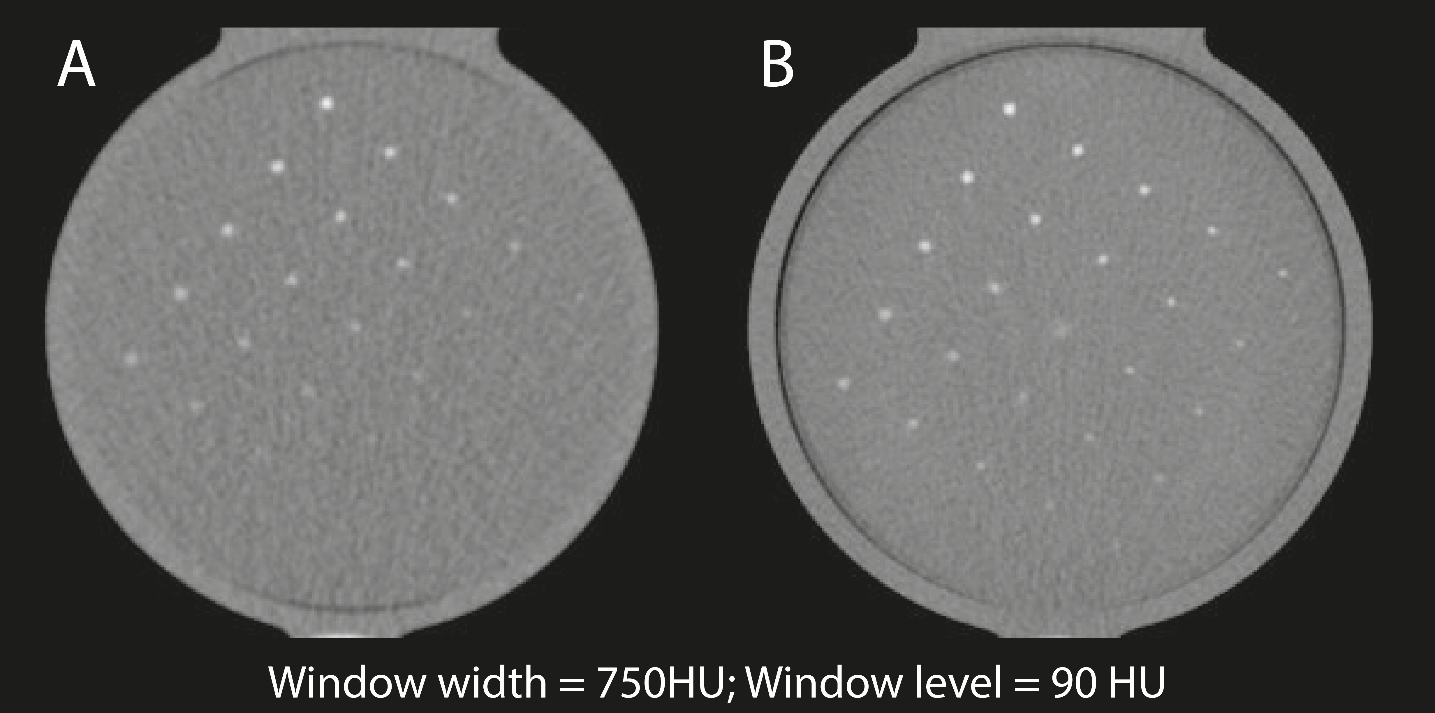


Supplemental Figure 3 Detectability curves for the small (A) and large (B) phantom with a 3 mm slice thickness. The red area indicates calcifications of different combinations of size and density which are not detected by both CT systems. Calcifications only detected with spectral photon-counting CT (SPCCT) are indicated in yellow. Green indicates that both systems detected a calcification


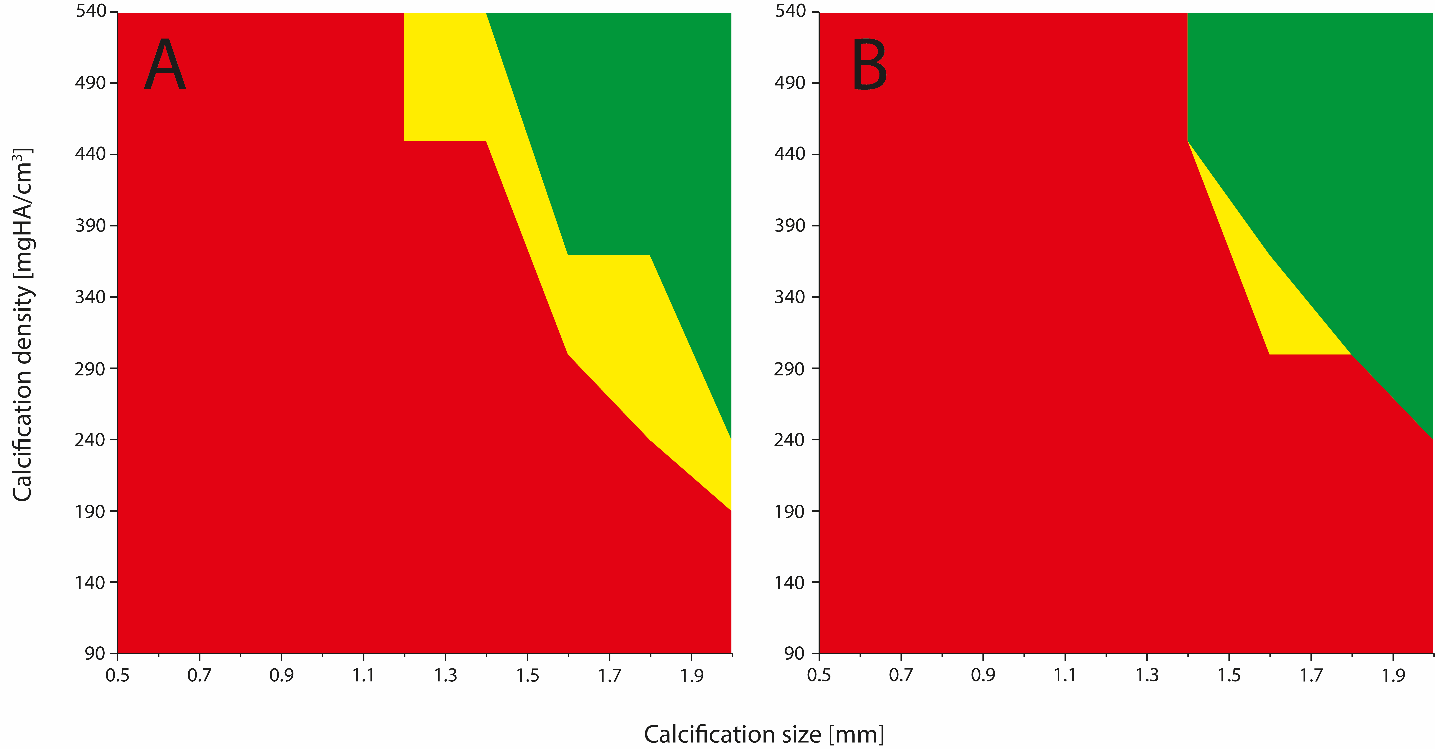

Supplement: Supplementary file 1 — (DOCX 753 kb) [file 330_2021_8152_MOESM1_ESM.docx]
